# Supplementary material for: The Effects of Lifestyle on COVID-19 Vaccine Hesitancy in the United States: An Analysis of Market Segmentation
Source: Int J Environ Res Public Health. 2022 Jun 24;19(13):7732. doi: 10.3390/ijerph19137732 (PMC9265792; doi:10.3390/ijerph19137732)
Supplement: Supplementary file 1 [file ijerph-19-07732-s001.zip › ijerph-1708405-supplementary.pdf]

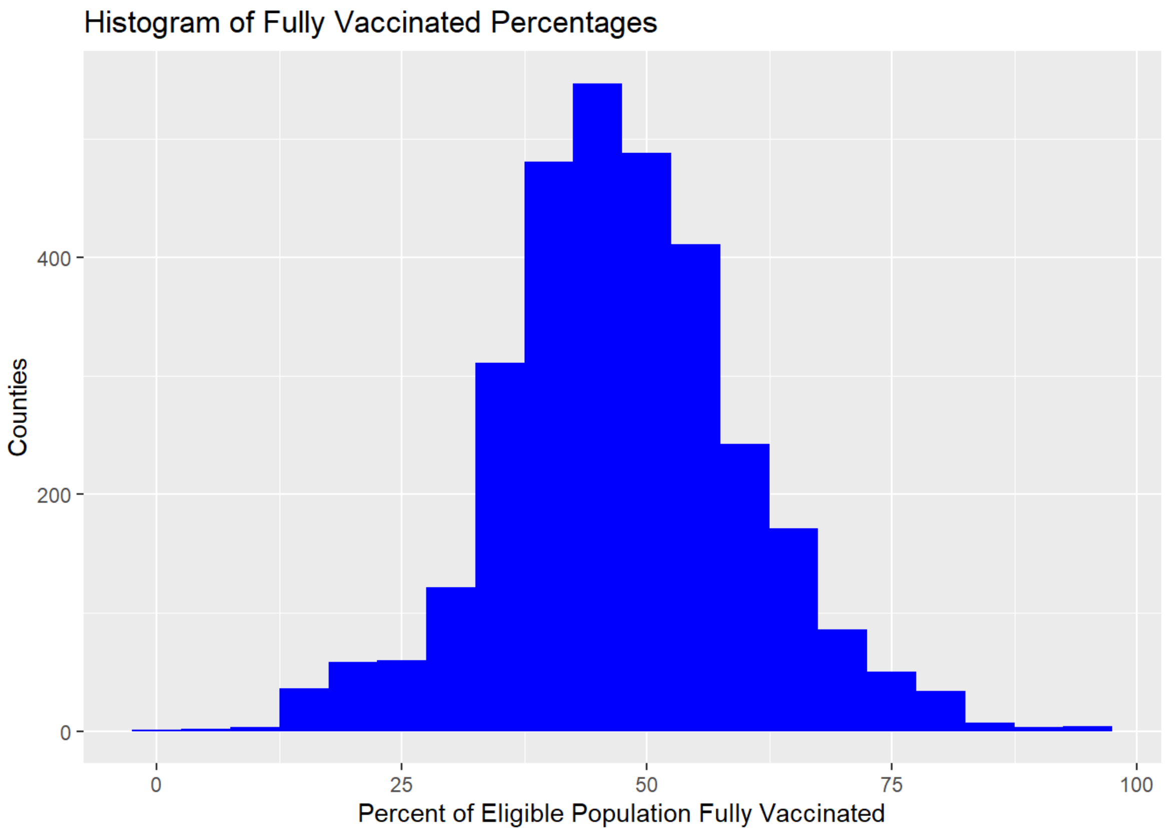

Figure S1. Histogram of county-by-county vaccination rates.

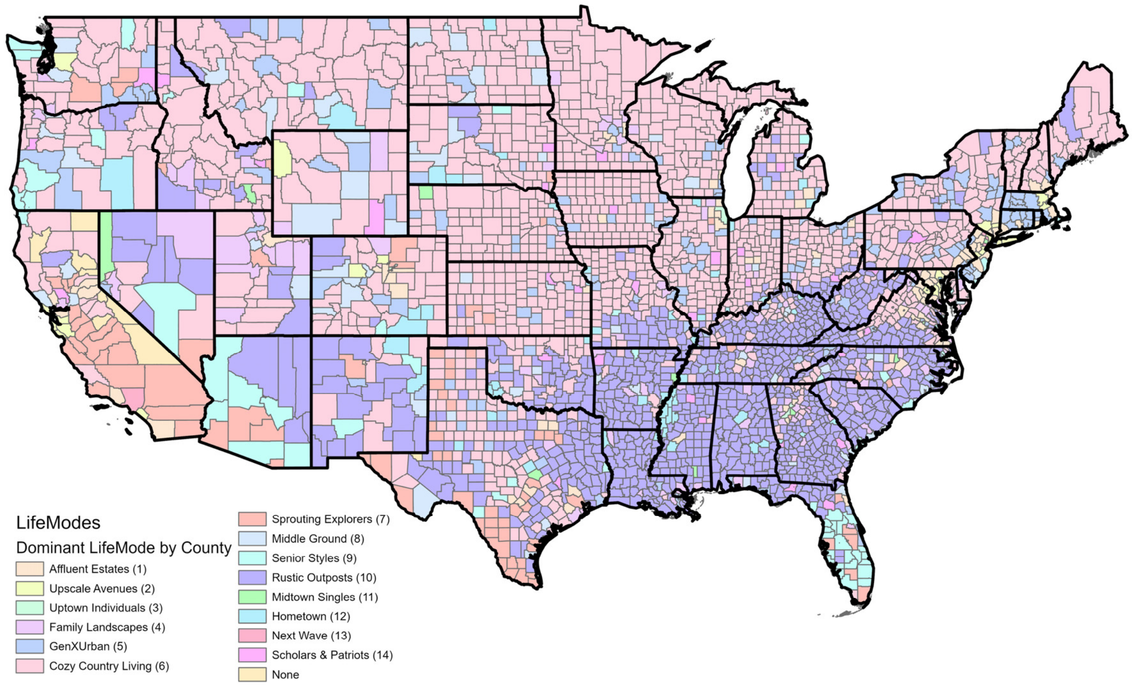

Figure S2. U.S. LifeModes.

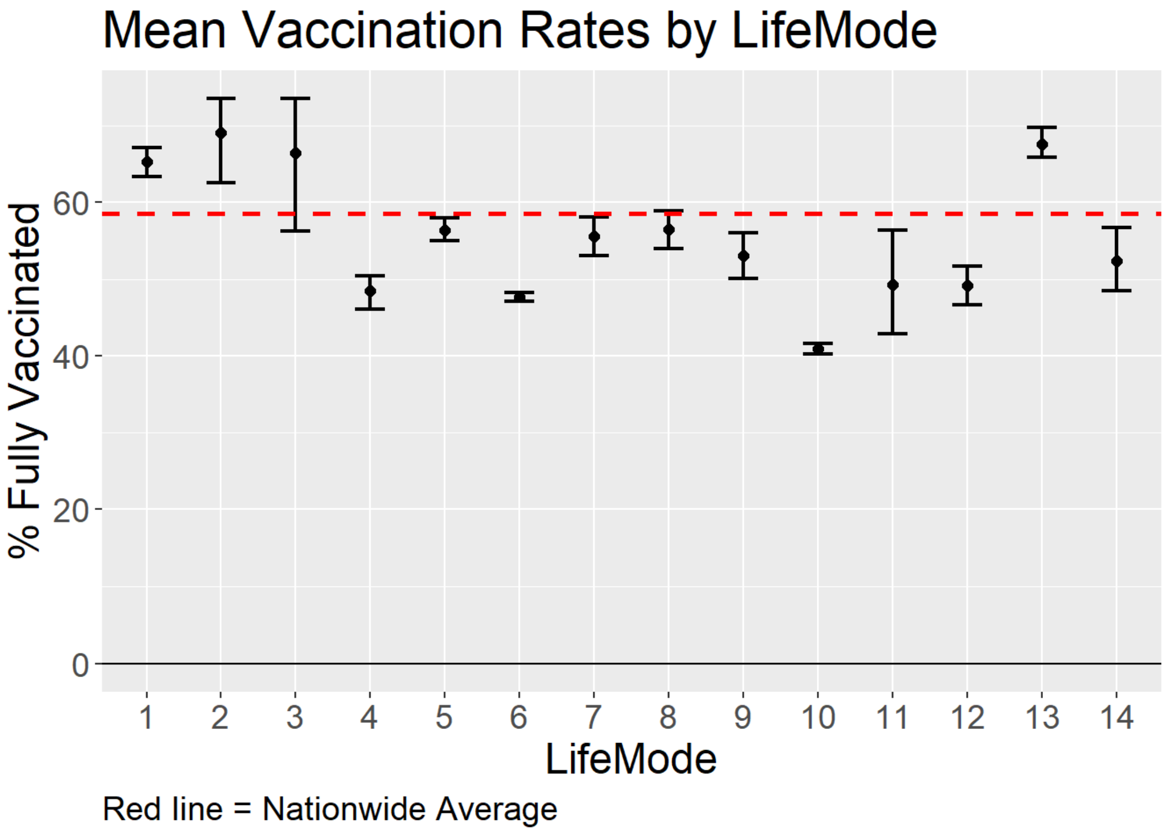

Figure S3. Mean Vaccination Rates by LifeMode

95% confidence intervals for the mean vaccination rate on January 1, 2022, for each of the fourteen LifeModes. The red dashed line denotes the nationwide mean for comparison.

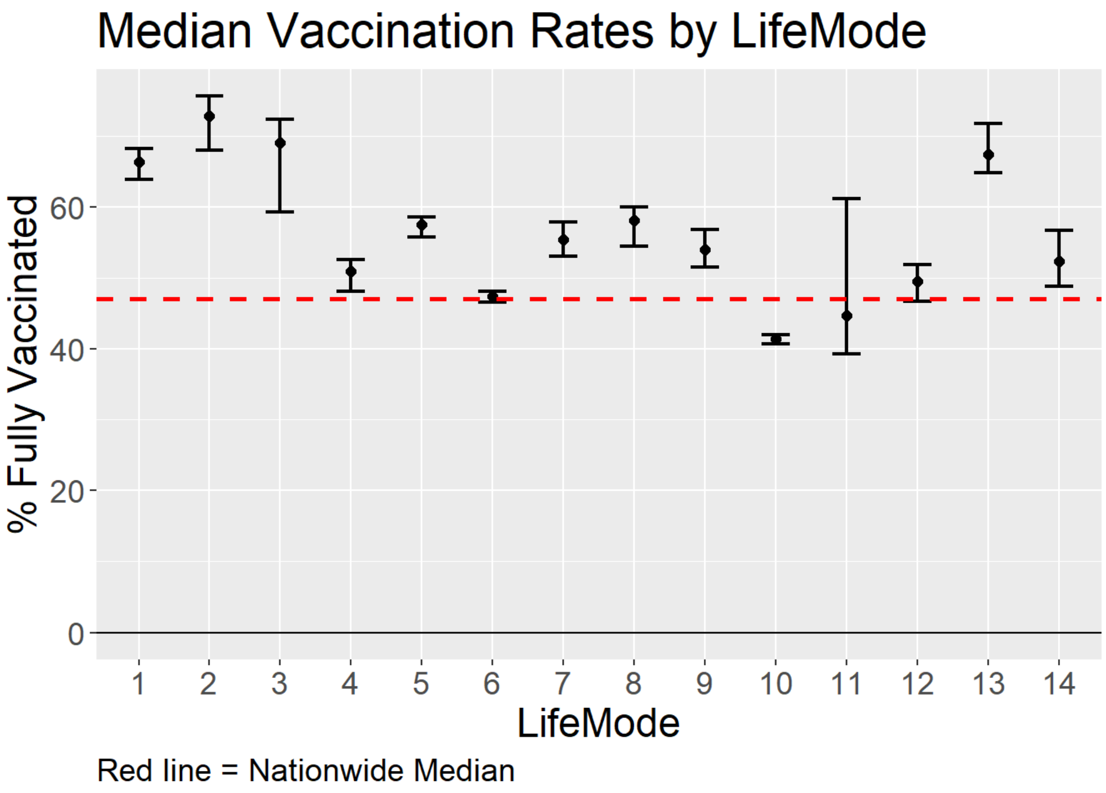

Figure S4. Median Vaccination Rates by LifeMode.

95% confidence intervals for the median vaccination rate on January 1, 2022, for each of the fourteen LifeModes. The red dashed line denotes the nationwide median for comparison.

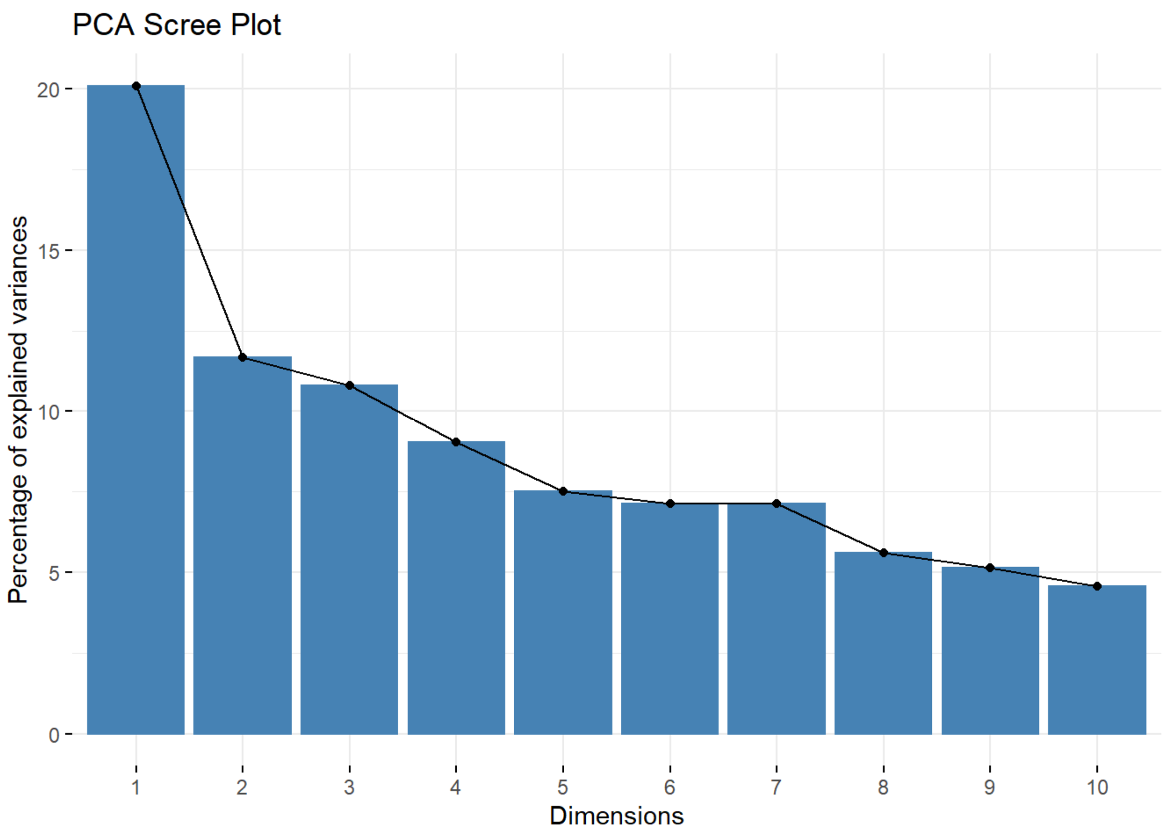

Figure S5. Principal Components Analysis (PCA) Scree Plot.
Scree plot showing the percentage of variance explained by each successive component in the PCA.
